# Retraction: Cross-cultural adaptation and psychometric testing of the Arabic version of the Modified Low Back Pain Disability Questionnaire

**DOI:** 10.1371/journal.pone.0268665

**Published:** 2022-06-09

**Authors:** Hamad S. Al Amer, Fahad Alanazi, Mohamed ELdesoky, Ayman Honin, the PLOS ONE Editors

Following publication of this article [1], it came to light that some of the content cannot be offered under the CC BY license used by PLOS journals.

The article [1] reports a translation of the Modified Low Back Pain Disability Questionnaire published in 2001 (referred to hereafter as MLBPDQ) [2, 3]. The MLBPDQ [2, 3] was originally modified from the Oswestry Low Back Pain Disability Questionnaire [4], which is licensed to the MAPI Trust [5].

A Correction to [2] published in 2008 [3] replaces Appendix 1 (the modified questionnaire) and amends the copyright and permission information associated with Appendix 1. At the time of publication of the *PLOS ONE* article [1], there was no notification on the web page for article [2] that a Correction had been published.

The authors of this article [1] have provided documentation to the *PLOS ONE* Editors to support that they took steps to request permission to publish a translated version of the modified questionnaire prior to publication of this article [1], including correspondence with the MAPI Trust and with RightsLink/Oxford University Press, which publishes PTJ on behalf of the American Physical Therapy Association. The corresponding author indicated that they were not aware of the correction to the MLBPDQ article [3] until after publication of [1]. In light of the Correction [3], the authors have made a revised permission request, but have been unable to obtain permission to include the translated modified questionnaire under the CC BY license.

In light of the removal of content that is not compatible with the CC BY license, the authors and the *PLOS ONE* Editors retract this article because it no longer meets the journal’s publication criteria.

The *PLOS ONE* Editors regret that this issue was not identified and resolved prior to publication and appreciate the efforts of the authors to address the concerns during the course of our follow up on this matter.

All authors agreed with retraction and stand by the article’s findings.

At the time of retraction, the Supporting Information files containing the translated MLBPDQ and S1 Table, are removed from the article. Furthermore, the retracted article has been amended throughout to remove the terms “Oswestry” and “MODQ” in reference to the Modified Low Back Pain Disability Questionnaire [2–3] and its translation, including amendment to the title and citation, removal of Figs 1–3, and removal of the peer review history. The article’s Data Availability statement and in-text citations of the relevant files have also been updated to reflect these changes.
